# Usefulness of Change in Estimated Glomerular Filtration Rate as a Predicting Factor of Progression of Chronic Kidney Disease

**DOI:** 10.5402/2013/351364

**Published:** 2012-11-26

**Authors:** Kunimi Maeda, Chieko Hamada, Satoshi Horikoshi, Yasuhiko Tomino

**Affiliations:** ^1^Division of Nephrology, Department of Internal Medicine, Juntendo University Nerima Hospital, 3-1-10 Takanodai, Nerima-ku, Tokyo 177-8521, Japan; ^2^Division of Nephrology, Department of Internal Medicine, Juntendo University School of Medicine, 2-1-1 Hongo, Bunkyo-ku, Tokyo 113-8421, Japan

## Abstract

*Purpose*. To explore factors contributing to chronic kidney disease (CKD) progression and change in estimated glomerular filtration rate over time (ΔeGFR) as a risk factor in predialysis patients under multidisciplinary managements. *Methods*. Among 113 CKD patients, eGFR, serum creatinine, total protein, albumin, urea nitrogen, uric acid, calcium, inorganic phosphate, total cholesterol, urinary creatinine, urinary protein (UP), hemoglobin A1c, hemoglobin, and hematocrit were analyzed. *Results*. ΔeGFR analysis in the first six months presented a positive slope (remission group) in 43 patients (38%) and a negative slope (no-remission group) in 70 patients (62%). Three-year dialysis-free rate was 89.4% in the remission group and 39.3% in the no-remission group, with a significant difference (*P* < 0.0001). To explore factors contributing to dialysis initiation by stepwise Cox regression, baseline eGFR (HR 0.706, *P* < 0.0001) and ΔeGFR in the first six months of treatment (HR 0.075, *P* < 0.0001) were identified. To investigate factors affecting remission and no remission by stepwise logistic regression, age (odds ratio 1.06, *P* = 0.018) and UP excretion (odds ratio 1.223, *P* = 0.045) were identified. *Conclusion*. Monitoring of ΔeGFR and UP is not only useful in suppressing CKD 3 progression, but also in deciding strategies to achieve remission in individual patients.

## 1. Introduction

In Japan, the number of patients with chronic kidney disease (CKD) shows a trend of annual increase and has reached 13.3 million as estimated by the Japanese Society of Nephrology. In other words, 1 in 9 adults is affected by CKD, showing a great impact of this disease on the population. In addition, the number of patients initiated on dialysis therapy continues to increase at a rate of 10,000 a year, reaching approximately 280,000 at the end of 2009 and has become an important medicoeconomic and social issue [1]. The Japanese Society of Nephrology tackled the establishment of standard treatment and dissemination of estimated glomerular filtration rate (eGFR) for the Japanese, which form the basis of CKD guidelines, and published the Clinical Practice Guidebook for Diagnosis and Treatment of Chronic Kidney Disease 2009 [2]. However, the current therapies cannot be regarded as optimal, and further studies of multidisciplinary treatment incorporating various modalities are required. 

With the previous background, further promotion of the use of eGFR as a simple indicator of the disease condition and practice of treatment based on evidence obtained from clinical studies are necessary. To investigate the relationship between the change in eGFR (ΔeGFR) with time and the suppression of CKD progression or delay of dialysis initiation, we conducted a retrospective research in patients at our clinic undergoing conventional therapies including antihypertensives (mainly renin-angiotensin system (RAS) blockers), erythropoietin, oral carbonaceous adsorbent (Kremezin), and dietary therapy and analyzed the factors that contribute to suppress CKD progression or delay dialysis initiation.

## 2. Patients and Methods 

The subjects of this study were selected from CKD patients attending the outpatient clinic of Juntendo University School of Medicine Nerima Hospital between January 2006 and January 2008, who were newly prescribed oral carbonaceous adsorbent (Kremezin, Kureha Corporation, Tokyo, Japan) in addition to their ongoing conventional treatments, and then followed continuously for more than three months. Patients who satisfied the following criteria were included: (1) having at least two data points of serum creatinine level within the first three months of treatment and (2) at least two data points of serum creatinine level between three to six months, with a total of at least 5 data points. A total of 113 patients were included in analysis (observation period 29 ± 10.4 months). The following clinical data were extracted: age, gender, primary disease, blood pressure, serum and urinary biochemical data, and concomitant medications. Estimated GFR for the Japanese was calculated by the modified version of Modification of Diet in Renal Disease equation [3]. Proteinuria was based on outpatient spot urine measurement. The baseline data was obtained 0 ± 14 days from the day of start of oral adsorbent therapy. 

Using the eGFR data obtained during the first six months of oral adsorbent therapy, a speed of CKD progression was calculated based on the slope of the regression line by the least square method. The patients were divided into two groups according to the slope: a positive slope (i.e., CKD improvement: remission group) and a negative slope (i.e., CKD progression: no-remission group). Next, to explore the risk factors of dialysis initiation, Cox regression analysis was conducted on 70 of 113 patients who had sufficient baseline data, and hazard ratios were calculated. Furthermore, a logistic regression analysis was performed to identify the factors associated with negative ΔeGFR in the first six months of treatment. 

## 3. Evaluations and Statistical Analysis 

For patient background at baseline, age and biochemical parameters are presented in mean ± standard deviation, and *t*-test was used in intergroup comparisons. The ΔeGFR is also presented in mean ± standard deviation. Dialysis-free rates were estimated by Kaplan-Meier method and differences between groups were analyzed by log-rank method. A *P* value less than 0.05 was considered statistically significant. Factors affecting dialysis-free rate and ΔeGFR were explored using Cox regression analysis and logistic regression analysis, respectively. Stepwise method was used to select variables in multivariate analyses. Statistical analyses were performed using SAS (Release 8.2, SAS Institute Inc., Cary, NC, USA). 

## 4. Results

### 4.1. Dialysis-Free Rate and Change in eGFR over Time

 The patient background and laboratory data of 113 patients analyzed in this study are shown in Table 1. The ΔeGFR in the first six months of treatment was −0.150 ± 0.816 mL/min/1.73 m^2^/month. The mean serum creatinine level of all patients was 3.0 ± 1.2 mg/dL. The majority of patients had stage 4 CKD, and stages 4 and 5 together accounted for 93% of all patients. The primary disease was diabetes in 38 patients (33.6%) and nondiabetic diseases in 75 patients (66.4%); the later included nephrosclerosis in 41 patients (36.3%), chronic nephritis in 16 patients (14.1%), and polycystic kidney disease in 6 patients (5.3%). The most frequent complication was hypertension (85.5%), followed by hyperuricemia (48.7%) and dyslipidemia (24.8%). As antihypertensives, angiotensin receptor blocker (ARB) was used by 70% and calcium antagonist by 60% of the patients. Dialysis-free rate in all patients was 86.6% at 12 months, 75.1% at 24 months, and 58.8% at 36 months. By CKD stage, the three-year dialysis-free rate was 87.5% in stage III, 76.6% in stage IV, and 29% in stage V. 

### 4.2. Comparisons between Remission Group versus No-Remission Group Based on ΔeGFR

**Figure 1:**
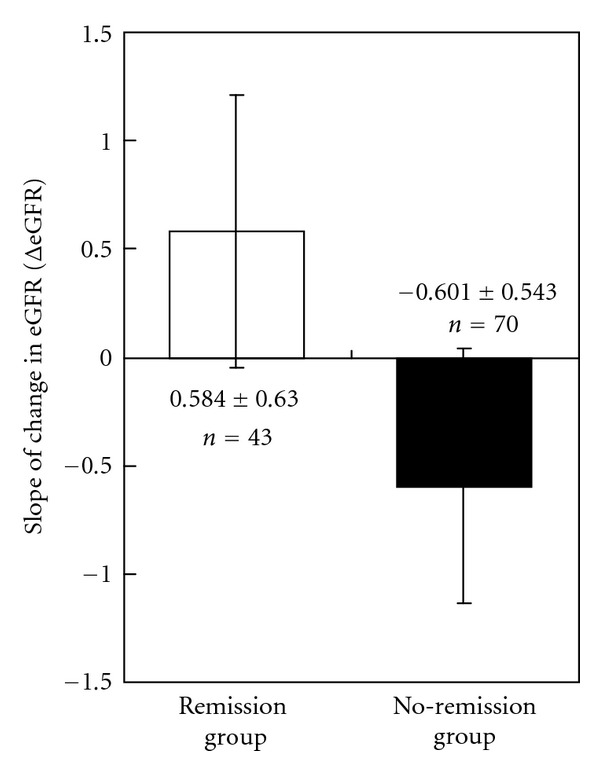
Change in estimated glomerular filtration rate (ΔeGFR) in six months in remission and no-remission groups.

Based on the eGFR data for the first six months of treatment, the slope of the regression line was calculated by the least square method, and 113 patients were divided into a positive slope group (i.e., eGFR increases) and a negative slope group (i.e., eGFR decrease). Among 113 patients, 43 (38%) had a positive slope (remission group: 0.584 ± 0.630 mL/min/1.73 m^2^/month) and 70 patients (62%) had a negative slope (no-remission group: −0.601 ± 0.543 mL/min/1.73 m^2^/month), showing suppression of CKD progression and improvement of eGFR in approximately 40% of the patients (Figure 1). In a comparison of patient background between two groups, gender, eGFR and urinary protein were significantly lower and hemoglobin and hematocrit were significantly higher in the remission group (Table 2).

**Figure 2:**
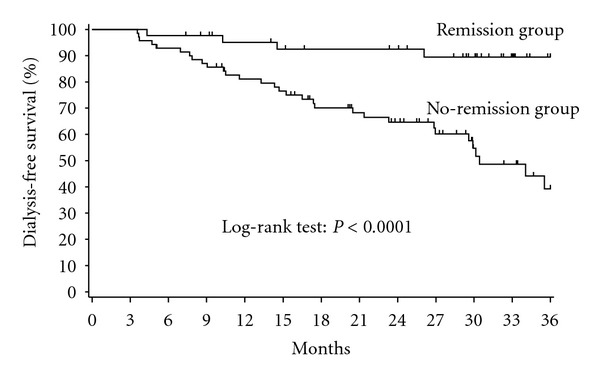
Dialysis-free survival rates in remission and no-remission groups.

Dialysis-free survival rates in the no-remission group (81% at 12 months, 64.6% at 24 months, and 39.3% at 36 months) were significantly lower than the corresponding rates in the remission group (95.1%, 92.5% and 89.4%, resp.; log-rank test: *P* < 0.0001) (Figure 2).

### 4.3. Risk Factors Associated with Dialysis Initiation

Cox regression analysis was conducted on 70 of 113 patients, who had all the required patient background and baseline laboratory data, to explore factors contributing to dialysis initiation (Table 3). Urinary protein and total cholesterol were described as a significant risk factor. Meanwhile, in the stepwise method, only baseline eGFR (hazard ratio 0.706, *P* < 0.0001) and ΔeGFR in the first six months of treatment (hazard ratio 0.075, *P* < 0.0001) were identified as significant factors associated with initiation of dialysis therapy. 

### 4.4. Analysis of Factors Affecting ΔeGFR

 A stepwise logistic regression analysis was conducted to examine the risk factors contributing to a negative ΔeGFR, that is, progression of CKD (Table 4). Age and urinary protein were identified as risk factors associated with CKD progression. 

## 5. Discussion 

In the present study, 43 of 113 patients (38%) had positive ΔeGFR (remission group: 0.584 ± 0.630 mL/min/1.73 m^2^/month) and 70 patients (62%) had negative ΔeGFR (no-remission group: −0.601 ± 0.543 mL/min/1.73 m^2^/month). Patients whose slope of change in eGFR with time showed a positive value had significantly lower urinary protein excretion and significantly higher hemoglobin and hematocrit. In this group of patients, dialysis-free survival rates also showed a significant delaying effect, confirming the relation between suppression of CKD progression and delay of dialysis initiation. Moreover, when the factors affecting CKD progression were examined using multivariate analysis, the result indicates that monitoring eGFR at the start of treatment and ΔeGFR during the first six months may predict progression of renal failure and initiation of dialysis. In other words, apart from the conventional risk factors of urinary protein, hemoglobin, and hematocrit, where baseline eGFR and ΔeGFR during treatment are also independent factors contributing to dialysis initiation. 

The present study had a long-term observation period of c.a. three years and analyzed factors that contribute to the outcomes of CKD treatment, namely, dialysis initiation which is a hard end point and ΔeGFR that indicates the speed of CKD progression. As far as we are aware, this is the first report demonstrating that suppressing CKD progression leads to prevent dialysis initiation using ΔeGFR. Advanced renal function impairment or proteinuria has been known to be risk factor for end-stage renal failure [4–7], while previous studies also showed that CKD per se is an independent risk factor of cardiovascular disease [8] and that the risk of cardiovascular death is much higher than the risk of end-stage renal failure in CKD [9]. Therefore, eGFR and proteinuria, the effecting factor and/or risk factors identified also in the present study, are important factors from the viewpoint of not only CKD but also cardiovascular events, and their importance is also recognized from the present results. 

In the study of patients at our institution, a positive ΔeGFR, or a trend of CKD improvement, was achieved after followup in approximately 40% of the patients. The possibility of remission of renal failure has been demonstrated by Sanaka et al. [10] in a retrospective study. In their study, multidisciplinary treatment including carbon adsorbent resulted in improvement in approximately one-third of patients with advanced chronic renal failure (mean serum creatinine 4.8 ± 1.9 mg/dL), although their patients had no hypoalbuminuria, hypercholesterolemia, hypertension, or anemia. In our remission group in which CKD progression was suppressed or stopped, baseline urinary protein was significantly lower and hemoglobin and hematocrit were significantly higher than the group showing no remission. As was also reported by Sanaka et al., lowering urinary protein and improving anemia are important to obtain therapeutic effect from CKD treatment. The result of multivariate analysis of factors associated with CKD progression indicates that monitoring eGFR at the time of treatment initiation and ΔeGFR during the first six months of therapy may predict the risk of dialysis initiation within three years. Since results from long-term longitudinal studies show a correlation between the rate of kidney function decline and the risk of death, Al-Aly and Cepeda [11] recommended to incorporate ΔeGFR in CKD staging to obtain a more dynamic classification. In the present study, ΔeGFR in the first 6 months of treatment correlates with dialysis initiation better than baseline GFR, supporting the importance of dynamic evaluation. Life-style-related diseases are involved in the onset and progression of CKD. While lifestyle modifications such as low-protein diet, as well as antihypertensive therapy (recently centering on angiotensin-converting-enzyme inhibitor (ACEi) and ARB), are conventionally used as CKD treatments, currently a multidisciplinary approach incorporating various modalities is being practiced. Although the renoprotective effect of ARB and ACEi has been reported in large-scale clinical trials such as RENAAL [12] and IDNT [13] conducted on CKD patients, the effects of oral adsorbent and dietary therapy have only been examined in small-scale trials [14–16] and therefore more evidence remains to be gathered. 

In the CAP-KD study, the mean baseline eGFR in the group receiving combination therapy with oral adsorbent was 22.2 ± 11.0 mL/min/1.73 m^2^, and the rate of eGFR change per year estimated by linear mixed model was −12% [17]. In the present study, the mean baseline eGFR in all patients was 18.6 ± 7.3 mL/min/1.73 m^2^ and ΔeGFR was −0.15 ± 0.816 mL/min/1.73 m^2^/month or a rate of eGFR change per year of approximately −10%. The overall ΔeGFR was less steep in our study despite a higher proportion of severe patients compared to the CAP-KD study, suggesting that regular monitoring of eGFR, urinary protein, and hemoglobin may contribute to improving the treatment outcome. The importance of maintaining good adherence to treatment based on renal function indicators, such as ΔeGFR and urinary protein, in suppressing the progression of renal dysfunction has been demonstrated in this study. For further development of multidisciplinary CKD treatment, the present study also demonstrated the importance of conducting treatment with continuous monitoring of ΔeGFR, an indicator that can be obtained simply at no extra cost by calculation from serum creatinine level, which allows patients to understand the treatment effect easily. Moreover, ΔeGFR appears to be a useful indicator for the evaluation of the therapeutic effect of oral adsorbent therapy, and a large-scale study is required to verify the present findings. 

## 6. Conclusion 

The present study investigated CKD progression and remission to treatment focusing on the change in eGFR with time. Even in patients with high-stage CKD, delay of dialysis initiation was achieved in those with a positive slope of eGFR change calculated from six-month data. Suppression of CKD progression is possible by using appropriate medical treatments while monitoring an indicator that is easily understood by patients. Furthermore, the present finding that ΔeGFR correlates with dialysis initiation demonstrates the importance of regular monitoring of eGFR. 

## Figures and Tables

**Table 1:** Basic characteristics of all patients.

Variables	No. analyzed	
Age; years	66.3 ± 13.3	
Gender; no. of patients (%)		
Female	38 (33.6%)	
Male	75 (66.4%)	
Stage; no. of patients (%)		
III	8 (7.1%)	
IV	62 (54.9%)	
V	43 (38.1%)	
Underlying disease; no. of patients (%)		
Diabetic	38 (33.9%)	
Non-diabetic	74 (66.1%)	
Systolic blood pressure; mmHg	140.6 ± 17.8	113
Diastolic blood pressure; mmHg	75.5 ± 13.2	113
eGFR; mL/min/1.73 m^2^	18.6 ± 7.3	113
Serum creatinine; mg/dL	3.0 ± 1.2	113
Total protein; g/dL	7.0 ± 0.7	110
Albumin; g/dL	3.9 ± 0.5	105
Urea nitrogen; mg/dL	39.9 ± 12.3	111
Uric acid; mg/dL	7.2 ± 1.7	111
Calcium; mg/dL	8.9 ± 0.7	103
Inorganic phosphate; mg/dL	3.9 ± 0.8	95
Total cholesterol; mg/dL	197.9 ± 45.6	101
Urinary protein; g/g·Cr	3.0 ± 3.2	81
Hemoglobin A1c; %	6.3 ± 1.4	57
Hemoglobin; g/dL	11.0 ± 2.0	110
Hematocrit; %	34.0 ± 5.6	110

Data are presented in means ± SD, unless stated otherwise. eGFR: estimated glomerular filtration rate.

**Table 2:** Basic characteristics of remission and no-remission groups.

		Remission group	No-remission group	*P *
Gender; no. of patients (%)				0.042
Female		9 (20.9%)	29 (41.4%)	
Male		34 (79.1%)	41 (58.6%)	
Age; years		65.8 ± 12.8 (*n* = 43)	68.6 ± 11.0 (*n* = 70)	0.221
Underlying disease; no. of patients (%)				0.421
Non-diabetic		31 (72.1%)	44 (62.9%)	
Diabetic		12 (27.9%)	26 (37.1%)	
CKD stage; no. of patients (%)				0.194
III		5 (11.6%)	3 (4.3%)	
IV		25 (58.1%)	37 (52.9%)	
V		13 (30.2%)	30 (42.9%)	
eGFR; mL/min/1.73 m^2^		20.8 ± 8.6 (*n* = 43)	17.3 ± 6.1 (*n* = 70)	0.026
serum creatinine; mg/dL		3.0 ± 1.5 (*n* = 43)	3.0 ± 1.0 (*n* = 70)	0.914
Total protein; g/dL		7.2 ± 0.7 (*n* = 41)	7.0 ± 0.7 (*n* = 69)	0.126
Albumin; g/dL		3.9 ± 0.6 (*n* = 39)	3.8 ± 0.5 (*n* = 66)	0.697
Urea nitrogen; mg/dL		38.7 ± 13.1 (*n* = 42)	40.6 ± 11.8 (*n* = 69)	0.433
Uric acid; mg/dl		7.4 ± 2.0 (*n* = 43)	7.0 ± 1.6 (*n* = 68)	0.239
Calcium; mg/dL		9.0 ± 0.9 (*n* = 39)	8.8 ± 0.6 (*n* = 64)	0.181
Inorganic phosphate; mg/dL		3.7 ± 0.8 (*n* = 35)	4.0 ± 0.8 (*n* = 60)	0.201
Total cholesterol; mg/dL		200.5 ± 44.8 (*n* = 38)	196.3 ± 46.3 (*n* = 63)	0.652
Urinary protein; g/g·Cr		2.1 ± 2.9 (*n* = 33)	3.6 ± 3.2 (*n* = 48)	0.036
Hemoglobin A1c; %		6.5 ± 1.8 (*n* = 23)	6.2 ± 1.2 (*n* = 34)	0.400
Hemoglobin; g/dL		11.6 ± 2.0 (*n* = 41)	10.7 ± 1.9 (*n* = 69)	0.013
Hematocrit; %		35.7 ± 5.7 (*n* = 41)	32.9 ± 5.3 (*n* = 69)	0.009

Data are presented in means ± SD, unless stated otherwise. eGFR: estimated glomerular filtration rate.

**Table 3:** Exploration of risk factors for dialysis initiation by Cox regression model.

Variable	Estimate	Prob-ChiSq	Hazard ratio	95% confidence interval
Male	1.1534	0.1095	3.169	0.772–13.01
Age	−0.0003	0.9921	1.000	0.944–1.058
eGFR	−0.4029	0.0001	0.668	0.545–0.820
ΔeGFR	−2.8075	<0.0001	0.060	0.018–0.202
Albumin	0.7558	0.3236	2.129	0.475–9.550
Uric acid	−0.0990	0.5902	0.906	0.632–1.299
Urinary protein	0.3864	0.0092	1.472	1.100–1.969
Inorganic phosphate	0.6224	0.1479	1.863	0.802–4.329
Total cholesterol	−0.0181	0.0234	0.982	0.967–0.998
Hemoglobin	−0.1055	0.6424	0.900	0.576–1.405
Diabetes	−0.8858	0.2171	0.412	0.101–1.683

eGFR: estimated glomerular filtration rate.

**Table 4:** Exploration of independent factors for decline of estimated glomerular filtration rate (i.e., progression of chronic kidney disease) by logistic regression analysis.

Variable	Estimate	PROB	Odds ratio	95% confidence interval
Age	0.0580	*P* = 0.018	1.060	1.010–1.112
Urinary protein	0.2015	*P* = 0.045	1.223	1.004–1.490
